# Mucosal Delivery of Recombinant SARS-CoV-2 Spike Receptor-Binding Domain Antigen Containing Immune-Stimulating Peptides Induces Protective Immune Responses Against Viral Infection in huACE2 Mice

**DOI:** 10.3390/vaccines14050421

**Published:** 2026-05-07

**Authors:** Byeol-Hee Cho, Ju Kim, Yong-Suk Jang

**Affiliations:** 1 Department of Bioactive Material Sciences and Research Center of Bioactive Materials, Jeonbuk National University, Jeonju 54896, Republic of Korea; 2 Korea Research Institute for Veterinary Biologics, Iksan 54531, Republic of Korea; byeolhee@krivb.or.kr; 3 Department of Molecular Biology and the Institute for Molecular Biology and Genetics, Jeonbuk National University, Jeonju 54896, Republic of Korea; ju226@jbnu.ac.kr

**Keywords:** cell-mediated immunity, human β-defensin 2, humoral immunity, M cell–targeting ligand, mucosal vaccine platform

## Abstract

**Background**: Severe acute respiratory syndrome coronavirus 2 (SARS-CoV-2) infects host cells through the interaction between the spike protein receptor-binding domain (RBD) and the human angiotensin-converting enzyme 2 (hACE2) receptor, which is expressed on epithelial cells in various tissues, including the respiratory tract. Therefore, mucosal immunity in the respiratory tract plays a key role in protection against viral infection. Previously, we demonstrated that intranasal administration of antigens (Ags) conjugated with the M cell-targeting peptide Co4B enhances both mucosal and systemic immune responses. That conjugation with human β-defensin 2 (HBD2) increases neutralizing antibody (Ab) responses. **Methods**: A recombinant antigen conjugate incorporating both Co4B and HBD2 was designed to enhance immunogenicity. Its immunogenicity was evaluated in mice following intranasal immunization. Antigen-specific antibody responses were measured in serum and bronchoalveolar lavage fluid. T-cell responses were evaluated in lungs and spleens. Protective efficacy was assessed using SARS-CoV-2-susceptible hACE2 knock-in mice. **Results**: Ag-specific Ab levels increased in both serum and bronchoalveolar lavage fluid of mice immunized intranasally with the conjugate. Especially, T-cell responses were significantly enhanced in the lungs and spleens of immunized hACE2 knock-in mice. In challenge experiments, intranasal administration of the conjugate reduced viral load. Moreover, Siglec F was identified as a potential receptor for Co4B, a previously uncharacterized M cell-targeting ligand. **Conclusions**: A recombinant viral Ag containing Co4B and HBD2 induces virus-specific humoral and cellular immune responses. Although further optimization of the vaccine formulation and administration strategy is needed, this conjugate shows potential as a platform for improving mucosal and systemic immunity.

## 1. Introduction

The development of vaccines for severe acute respiratory syndrome coronavirus 2 (SARS-CoV-2) has progressed more rapidly than for any other viral vaccine. The mRNA vaccines encoding the SARS-CoV-2 spike protein were commercialized after establishing their efficacy in preventing infection [1,2]. Additionally, recombinant SARS-CoV-2 spike protein vaccines have demonstrated protective efficacy against the virus [3]. However, the various SARS-CoV-2 vaccines are not effective in inducing mucosal immunity when they are administered via intramuscular injection [4,5]. Nevertheless, most vaccines for respiratory viruses, including SARS-CoV-2, continue to be administered intramuscularly. Mucosal immunity plays a critical role in preventing viral infections at mucosal surfaces and in generating memory T-cells and B-cells in the lungs [4,5]. In particular, antigen (Ag)-specific secretory immunoglobulin (Ig) A, a key component of mucosal immunity, is secreted at the respiratory mucosa, where it neutralizes viruses and limits respiratory viral infections. Intranasal vaccination has been shown to elicit mucosal immune responses that protect the respiratory tract—the main site of infection—and create a barrier against viral pathogens [6]. Furthermore, nasal vaccination can generate both respiratory mucosal and systemic immune responses to prevent viral infections [7]. Consequently, nasal vaccination is a promising strategy for preventing viral infections, and further research is needed to develop effective nasal mucosal vaccines against respiratory viruses.

Currently, five mucosal vaccines for SARS-CoV-2—Convidecia Air^®^, iNCOVACC^®^, Gam-COVID-Vac, Pneucolin^®^, RAZI-COV PARS—administered via inhalation or intranasally have received approval for human use in China, Morocco, Indonesia, India, Russia, or Iran [8]. In addition, approved intranasal vaccines consist of a live attenuated vaccine for influenza A and B, as well as a recombinant virus-like particle vaccine for chronic hepatitis B [9]. Additionally, preclinical studies are actively investigating nasal vaccines for other pathogens that infect via mucosal surfaces, including respiratory syncytial virus, human immunodeficiency virus-1, tuberculosis, and SARS-CoV-2 [10,11,12,13]. To overcome the obstacles presented by the viscous mucus environment, which limits the efficacy of nasally delivered immunogens, nasal vaccines employ a range of delivery methods, including liposomes, particulate carriers, nanoparticles, virus-like particles, and emulsions [9,14]. One promising approach for enhancing the delivery of Ags to the mucosal immune system targets microfold (M) cells. M cells are specialized cells in the subepithelial domes of Peyer’s patches (PPs) and nasal-associated lymphoid tissue (NALT) that play a key role in Ag uptake [15]. Positioned at the mucosal surface and continuously exposed to the external environment, M cells facilitate the transcytosis of Ags into NALT [16]. Interaction between a Ag and a protein specifically expressed in M cells enhances both the introduction of the Ag and the subsequent Ag-specific immune response [17]. In previous studies, we identified M cell-targeting ligands, such as the Co4B peptide, using biopanning of a phage display library against M cell-like cells differentiated in vitro; this peptide binds specifically to M cells and enhances antigen uptake [18]. The ligands demonstrated efficacy for nasal immunization, and intranasal administration of the toxin Ag ApxIIA conjugated with Co4B elicited protective immune responses against *Actinobacillus* *pleuropneumoniae* [18]. Additionally, we demonstrated that intranasal administration of the receptor-binding domain (RBD) of the Middle East respiratory syndrome coronavirus (MERS-CoV) spike protein conjugated to Co4B increased protective immune responses against MERS-CoV in human dipeptidyl peptidase 4-transgenic mice, which are susceptible to MERS-CoV infection [19]. However, the specific components of the apical membrane of M cells that might function as potential receptors for Co4B and the immune responses regulated by Co4B remain uncharacterized.

Human β-defensin 2 (HBD2) is an antimicrobial peptide (AMP) secreted by epithelial cells, including those in the skin, lungs, and nasal mucosa [20,21,22]. HBD2 is recognized for triggering innate immune responses against numerous pathogens, such as bacteria, viruses, and fungi, thereby protecting against infection [23,24,25]. Additionally, HBD2 plays a role in adaptive immune responses by recruiting monocytes, dendritic cells, neutrophils, and T-cells [26,27,28,29]. Previous research has demonstrated that HBD2 can induce type I interferon (IFN) expression and inflammatory responses through Nod2 signaling in macrophages, thereby enhancing Ag-specific adaptive immune responses to a Ag internalized via HBD2 [30,31]. In mice immunized intranasally with the RBD of the MERS-CoV spike protein conjugated with HBD2, Ag-specific antibody (Ab) responses were significantly higher than they were in mice immunized via intramuscular injection [31,32]. Consequently, HBD2 is considered an effective adjuvant peptide that supports both innate and adaptive immune responses following nasal immunization.

In this study, we aimed to develop an intranasal mucosal vaccine platform that can elicit effective immune responses against respiratory mucosal pathogens. To construct the platform, we conjugated HBD2 and Co4B, both of which are known to enhance mucosal immune responses, to the N and C-termini of the SARS-CoV-2 spike RBD (RBD19), respectively. We demonstrated that the HBD2-RBD19-Co4B conjugate enhanced Ag-specific immune responses both locally and systemically when administered intranasally in mice. Furthermore, we found that this conjugate provided effective protection against SARS-CoV-2 in a challenge experiment using human angiotensin-converting enzyme 2 (hACE2) knock-in (KI) mice, which are susceptible to SARS-CoV-2 infection. Additionally, we identified Siglec F, a receptor molecule expressed on M cells, as a potential binding partner for Co4B. Our findings suggest that vaccine constructs containing HBD2 and Co4B could serve as a mucosal vaccine platform that induces both mucosal and systemic immune responses upon nasal administration.

## 2. Materials and Methods

### 2.1. Reagents and Laboratory Apparatus

All chemical reagents were obtained from Sigma Chemical Co. (St. Louis, MO, USA), except where noted otherwise. Experimental consumables were mainly from SPL Life Sciences (Pyeongtaek, Republic of Korea). Oligonucleotide primers were synthesized by Bioneer Inc. (Daejeon, Republic of Korea).

### 2.2. Experimental Animals and Virus Preparation

Female C57BL/6 and BALB/c mice (7 weeks old, weight around 20–25 g; Koatech Laboratory Animal Center, Pyeongtaek, Republic of Korea) and hACE2-All CDS B6J KI mice (7–8 weeks old, weight around 20–30 g; Cyagen, Santa Clara, CA, USA) were maintained in a sterile environment with unrestricted access to food and water. hACE2-All CDS B6J KI mice express human ACE2 under endogenous regulatory elements [33]. Unlike the K18-hACE2 mouse model, which exhibits systemic overexpression, these models have been reported to exhibit physiological expression patterns and infection patterns similar to those observed in humans [34]. After a 7-day acclimation period, mice with extremely low body weight or poor coat condition were excluded from in vivo experiments. Health status was verified, including factors that could affect experimental results, and mice were randomly assigned to groups of 3 or 6. Procedures involving animals followed ethical guidelines and the approved standards of Jeonbuk National University’s Institutional Animal Research Ethics Committee (JBNU 2022-081, JBNU 2022-082). Details of the study design and experimental procedures were provided in accordance with the ARRIVE guidelines to ensure reproducibility. The SARS-CoV-2 (KCDC03-NCCP 43326/2020) was acquired from the National Culture Collection for Pathogens (Cheongju, Republic of Korea). Virus-related experiments were performed in a biosafety level 3 isolation facility at the Korea Zoonosis Research Institute, Jeonbuk National University (Iksan, Republic of Korea), in accordance with the World Health Organization’s recommendations.

### 2.3. Genetic Engineering and Protein Production

**Figure 1:**
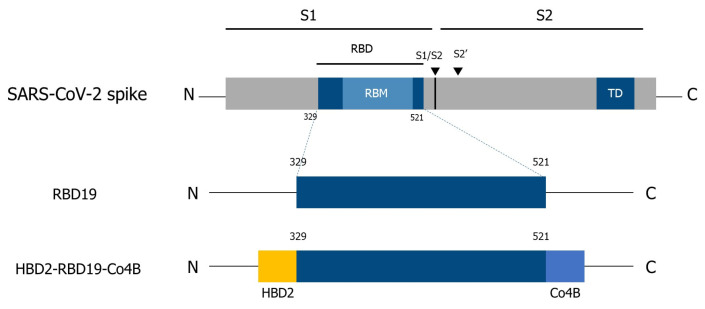
Schematic diagram for the construction of recombinant antigen proteins using the receptor-binding domain of the spike protein of SARS-CoV-2. RBD, receptor-binding domain; RBM, receptor-binding motif; TD, transmembrane domain.

The spike RBD of SARS-CoV-2 (Wuhan-Hu-1, YP_009724390.1) was fused with HBD2 (AF071216.1) at the N-terminal end and Co4B (HTLGFTVPTHAK) at the C-terminal end (Figure 1). The RBD sequence used to generate the recombinant antigen was derived from the Wuhan strain of SARS-CoV-2 and is identical to that of the viral strain used in the neutralization assays and in vivo challenge experiments. The recombinant gene coding the RBD fused with HBD2 and Co4B was synthesized by Bioneer Inc. (Daejeon, Republic of Korea). The *RBD19* gene was generated from the HBD2-RBD19-Co4B template through amplification with the following forward and reverse primers: 5′-CAT ATG *GAG CTC* ATG TTT CCT AAT ATT ACA AAC-3′ (SacI restriction site in italics) and 5′-GC*T CTA GA*T TAT GGT GCA TGT AGA AGT TC-3′ (XbaI restriction site in italics), respectively. To create recombinant EGFP-Co4B and EGFP proteins, the *EGFP* gene (Sequence ID: JQ693016.1) was first synthesized by Bioneer Inc. The recombinant gene encoding EGFP-Co4B was amplified from the *EGFP* gene template using the following forward and reverse primers: 5′-C*GA GCT C*AT GGT GAG CAA GGG CGA G-3′ (SacI restriction site in italics) and 5′-G*CT CTA G*AT TAC TTA GCA TGA GTA GGA ACC GTA AAA CCC AGC GTT GCT TGT ACA GCT CGT C-3′ (XbaI restriction site in italics), respectively. The recombinant gene construct was ligated into the pCold II vector and expressed using the *E. coli* expression system. Recombinant proteins were isolated via Ni-NTA agarose (Qiagen, Hilden, Germany), achieving >90% purity. Endotoxin levels (<0.5 EU/μg) were validated using an LAL chromogenic assay (Thermo Fisher Scientific, Waltham, MA, USA).

### 2.4. Immunization Protocol and Viral Challenge

C57BL/6 and hACE2 KI mice were intranasally immunized once weekly for five weeks with 10 μg of either RBD19 or HBD2-RBD19-Co4B recombinant protein under anesthesia. Phosphate-buffered saline (PBS) was administered as a negative control. Mice were immunized once weekly for five weeks, followed by a booster dose of 10 μg of the corresponding Ag or PBS administered two months later. To evaluate Ag-specific Ab responses, serum samples were collected three days post-boost. Bronchoalveolar lavage fluids (BALFs) were harvested seven days after the booster immunization. At the same time, spleens, lungs, and NALTs were collected for analysis of Ag-specific T-cell responses. Sample processing and preparation followed previously established protocols [35].

For the viral challenge experiments, SARS-CoV-2 (KCDC03-NCCP 43326/2020) was propagated in Vero E6 cells with Dulbecco’s modified Eagle medium containing 10% fetal bovine serum. The cells were incubated with the virus at a multiplicity of infection of 0.1 or less and incubated for 48 h before harvesting the virus-containing supernatant. Seven days following the booster immunization, hACE2 KI mice were intranasally challenged with 2 × 10^5^ plaque-forming units (PFUs) of SARS-CoV-2, a dose expected to induce approximately 50% mortality based on previous studies [34,36]. Post-infection, the mice were observed daily for survival, body weight changes, and clinical signs for at least 8 days. Infected mice were sacrificed at 8 days post-infection (dpi), and tissue specimens were collected at random. Some tissues were used to quantify viral RNA by real-time quantitative reverse transcription PCR (qRT-PCR).

To reconfirm the experimental results, immunization and viral challenge experiments were independently repeated with separate animal groups. The entire study, including these experiments, was repeated in independent biological replicates and presented with representative results from duplicate experiments.

### 2.5. Ag-Specific Ab Response Analysis

Enzyme-linked immunosorbent assay (ELISA) plates (Corning, NY, USA) were prepared by coating each well with 200 ng of RBD19 protein diluted in coating buffer and incubated overnight at 4 °C. After the plates were blocked, serial dilutions of serum or BALF samples were dispensed into each well and incubated overnight at 4 °C. Bound Ag-specific Abs were detected using alkaline phosphatase (AP)-conjugated secondary Abs. Colorimetric development was performed by adding the appropriate substrate, and absorbance was measured with a SPECTROstar Nano microplate reader (BMG LABTECH, Ortenberg, Germany).

To investigate interactions between Co4B and Siglec F, recombinant EGFP-Co4B, EGFP alone, and Co4B peptide alone (Mimotopes, Mulgrave, VIC, Australia) were individually coated onto ELISA plates. After the plates were blocked, Siglec F protein (R&D Systems, Minneapolis, MN, USA) was incubated with the coated proteins or peptides for 2 h. The wells were then treated with anti-CD170 (Siglec F) Ab (eBioscience, San Diego, CA, USA) or anti-His Ab (Qiagen) for 2 h at room temperature, followed by treatment with AP-conjugated secondary Abs. Color development and intensity measurement were conducted as described above.

### 2.6. Viral Neutralization Assay

Vero E6 cells were seeded and incubated overnight in 96-well plates at a density of 1.5 × 10^4^ cells per well. Sera from immunized mice were diluted at 1:20 and mixed with SARS-CoV-2 (KCDC03-NCCP 43326/2020, 90 focus-forming units/well). After the culture medium was removed, the cells were incubated with virus-serum mixtures for 1 h at 37 °C. Subsequently, overlay medium was dispensed onto the plates, and the plates were incubated for 24 h at 37 °C. After fixation with a 1:1 methanol-acetone mixture for 15 min at room temperature, blocking was conducted overnight at 4 °C using 5% skim milk. The cells were then exposed to anti-SARS-CoV-2 RBD (Invitrogen, Waltham, MA, USA) and spike (ProSci Incorporated, Poway, CA, USA) protein Abs at room temperature for 2 h, followed by treatment with horseradish peroxidase-conjugated anti-rabbit IgG Ab. Foci were detected using KPL TrueBlue™ substrate (Seracare, Milford, MA, USA) and analyzed.

### 2.7. Cellular Immune Response Analysis

Spleens and lungs were collected from immunized mice and mechanically dissociated. Tissue suspensions were enzymatically processed with collagenase and DNase I. Lymphocytes were isolated by Percoll density gradient centrifugation (Cytiva, Marlborough, MA, USA). The cells were treated with 1 μg of RBD19 protein for 24 h. For intracellular cytokine staining of IFN-γ and granzyme B (GrB), GolgiPlug™ and GolgiStop™ (BD Biosciences, Franklin Lakes, NJ, USA) were introduced during the last 5 h of incubation. After rat anti-mouse CD16/32 Ab (Invitrogen) was applied to block the Fc receptor, the cells were stained using fluorochrome-linked Abs targeting CD3, CD4, CD8, and CD44 (BioLegend, Miltenyi Biotec, Bergisch Gladbach, Germany). The lymphocyte population was gated for CD3^+^ cells, then divided into CD4^+^ and CD8^+^ subsets, with individual gates set for further analysis (Figure S4). For intracellular cytokine detection, the cells were fixed and permeabilized with BD Cytofix/Cytoperm™ reagent and stained with anti-IFN-γ and anti-GrB Abs. Flow cytometry was performed using a CytoFLEX flow cytometer (Beckman Coulter, Brea, CA, USA).

### 2.8. Viral Load Quantification

Lung RNA from infected mice was homogenized in TRIzol (Thermo Fisher Scientific), reverse-transcribed (Promega, Madison, WI, USA), and analyzed by qRT-PCR (CFX Connect System; Bio-Rad, Hercules, CA, USA). The PCR conditions consisted of an initial 95 °C denaturation for 3 min followed by 40 cycles of 95 °C (15 s), 52 °C (30 s), and 72 °C (30 s). The primer sequences were as follows: N protein of SARS-CoV-2, forward 5′-ATG CTG CAA TCG TGC TAC AA-3′, reverse 5′-GAC TGC CGC CTC TC-3′ [37]; mouse β-actin, forward 5′-CGT ACC ACA GGC ATT GTG A-3′, reverse 5′-CTC GTT GCC AAT AGT GAT GA-3′ [38]. Relative expression levels were normalized to β-actin, and the fold changes were calculated using CFX Maestro 1.0 software (Version: 4.0.2325.0418, Bio-Rad).

### 2.9. Tissue Localization Analysis

EGFP-Co4B (10 μg) was administered intranasally to BALB/c mice. EGFP not conjugated with Co4B was administered as a control. NALT sections were sliced via a cryo-microtome (Thermo Fisher Scientific), fixed, and stained with anti-Siglec F-Alexa 647 (BD Bioscience) and anti-EGFP-Alexa 488 (Invitrogen, Waltham, MA, USA). Images were acquired using a FLUOVIEW™ FV3000 (Olympus, Tokyo, Japan).

To identify functional receptors interacting with Co4B, 10 μg of EGFP-Co4B was intranasally administered to 7-week-old BALB/c mice. At 1 h post-administration, NALTs were collected and sectioned using a cryostat microtome (HM525 NX, Thermo Fisher Scientific). Fixation was performed using 4% paraformaldehyde solution for 15 min at room temperature. For PPs, fixation was performed for 24 h, and then they were incubated with 1 μg of EGFP-Co4B for 2 h. After they were blocked, the samples were treated overnight at 4 °C with anti-M cell Ab (Miltenyi Biotec, Bergisch Gladbach, Germany). Secondary staining comprised anti-rat IgG-Alexa 680, anti-Siglec F-Alexa 647, and anti-EGFP-Alexa 488 Abs. DAPI was used to counterstain the nuclei, and antifade mounting medium was applied to the samples. Imaging was performed using a FLUOVIEW™ FV3000 confocal laser scanning microscope (CLSM, Olympus), and images were analyzed with FV31S-SW software (Version: 2.6.1.243).

### 2.10. Statistical Analysis

**Figure 2:**
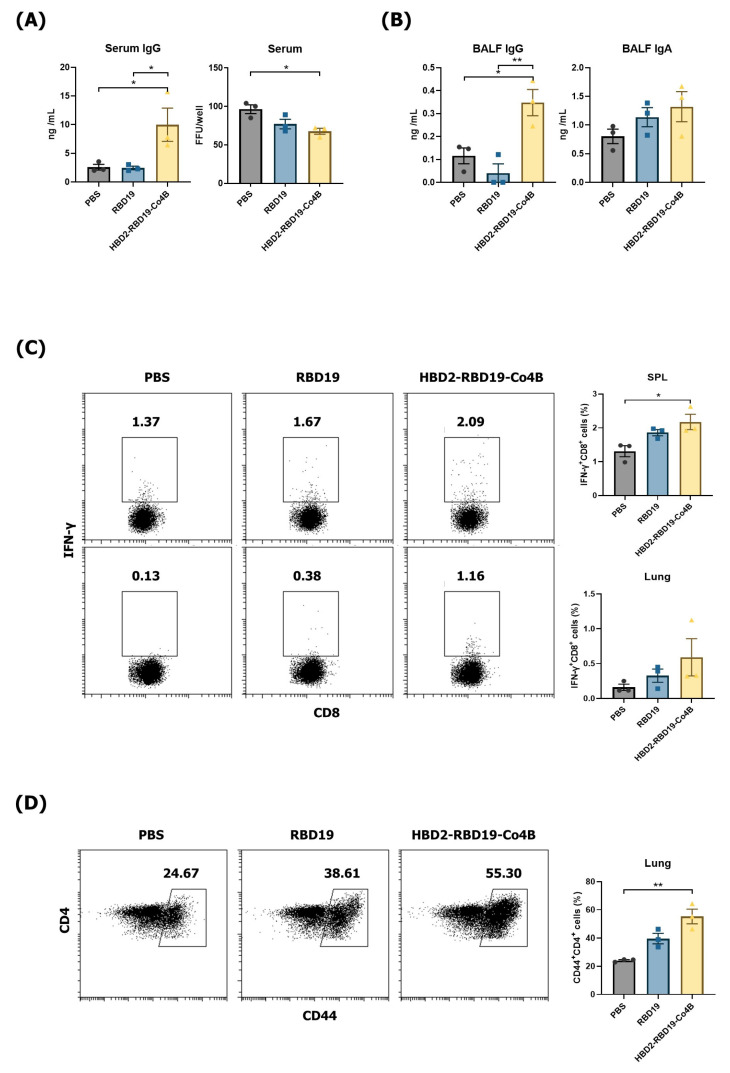
Induction of Ag-specific Ab and T-cell immune responses in C57BL/6 mice following booster immunization with recombinant RBD19 proteins. (**A**) The levels of Ag-specific Abs were measured by ELISA in sera collected 3 days after the boost immunization. Then, the levels of virus-neutralizing Ab were measured via a focus reduction neutralization assay, as described in the Materials and Methods. (**B**) The levels of Ag-specific IgG and IgA in BALF collected from C57BL/6 mice 7 days after the boost immunization. (**C**) IFN-γ^+^CD8^+^ T-cells were detected in lungs and spleens using flow cytometry. (**D**) Persistence of CD44^+^CD4^+^ T-cells in the lungs was observed for 2 months. Flow cytometry graphs show data from representative individual mice. Bar graphs present pooled data, with each dot representing results from an individual mouse. Data are shown as the mean ± standard error (SE) of at least two repeated experiments. All data were analyzed by one-way ANOVA (* *p* < 0.05, ** *p* < 0.01, *n* = 3).

**Figure 3:**
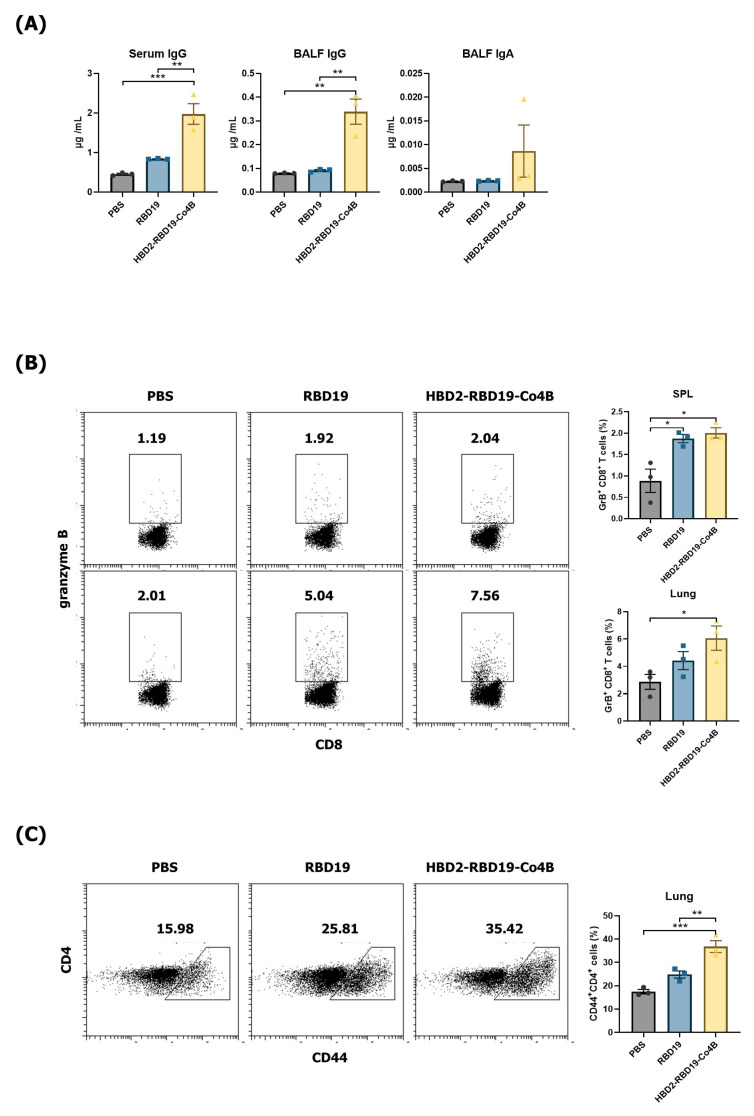
RBD19-specific Ab and T-cell responses 2 months after the immunization of hACE2 KI mice. (**A**) The level of Ag-specific IgG in immunized hACE2 KI mice was measured in sera collected 3 days after the boost immunization, and IgG and IgA were measured in BALF collected 7 days after the boost immunization, all with ELISA. (**B**) GrB^+^ CD8^+^ T-cells were detected in splenocytes and lung lymphocytes using flow cytometry. (**C**) CD44^+^CD4^+^ T-cells were maintained in the lungs for 2 months. Flow cytometry graphs show data from representative individual mice. Bar graphs present pooled data, with each dot representing results from an individual mouse. Data represent the mean ± SEs of at least two repeated experiments, and were analyzed by one-way ANOVA (* *p* < 0.05, ** *p* < 0.01, *** *p* < 0.001, *n* = 3).

**Figure 4:**
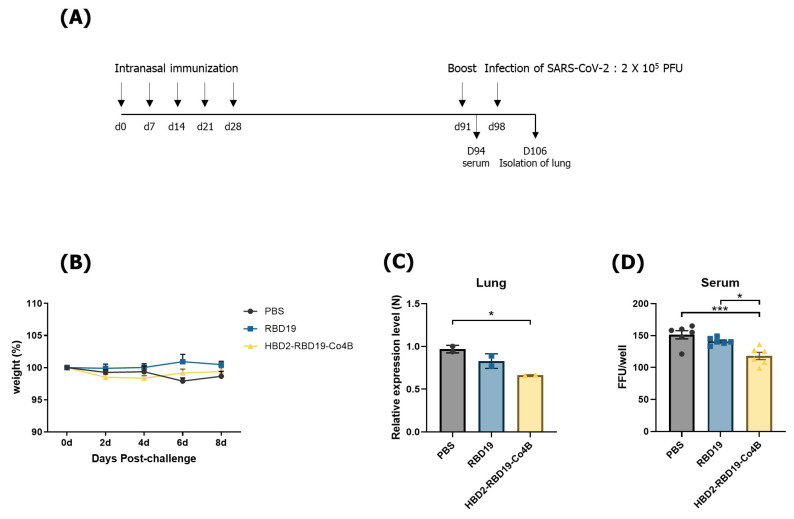
SARS-CoV-2 infection in hACE2 KI mice was inhibited by intranasal immunization with the conjugate Ag. (**A**) Schedule for immunization and challenge infection. (**B**) Body weight changes in mice (*n* = 6) after infection with SARS-CoV-2. (**C**) Level of SARS-CoV-2 detected by qRT-PCR in lungs isolated 8 days after the challenge infection (*n* = 2), as described in the Materials and Methods. (**D**) The neutralization effect was determined using sera and a focus-forming assay. Data are presented as mean ± SEM or SD from at least two independent experiments. Statistical significance was determined using one-way or two-way ANOVA (* *p* < 0.05, *** *p* < 0.001).

**Figure 5:**
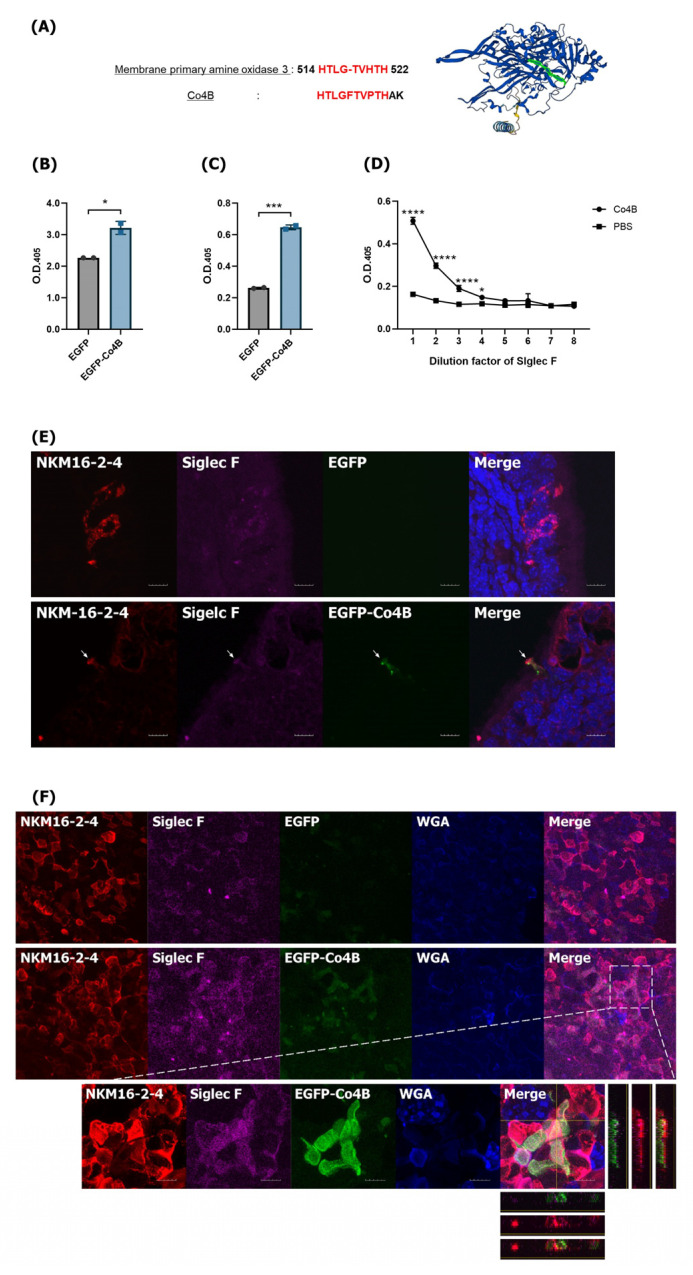
Interaction between Co4B and Siglec F. (**A**) Similarity between the Co4B and VAP-1 amino acid sequences. (**B**) EGFP and EGFP-Co4B were incubated on plates coated with recombinant Siglec F, and binding was detected using ELISA. (**C**) Binding of recombinant Siglec F to immobilized EGFP–Co4B was quantified by ELISA. (**D**) Binding of Siglec F to immobilized Co4B peptides was assessed by ELISA using serial dilutions of Siglec F. (**E**) NALTs were collected and sectioned after administration of EGFP-Co4B. Colocalization of EGFP-Co4B and Siglec F on M cells (identified using NKM 16-2-4, red) in NALT was assessed based on fluorescence signal overlap. Scale bars represent 50 μm. (**F**) The expression of Siglec F (purple) on M cells (green) in a Peyer’s patch (PP) was visualized by confocal laser-scanning microscopy, demonstrating colocalization of EGFP-Co4B with Siglec F on M cells. Scale bar represents 10 μm. Data represent the mean ± SDs of at least two independent experiments. Statistical significance in (**B**,**C**) was determined using an unpaired *t*-test, and in (**D**) using two-way ANOVA (* *p* < 0.05, *** *p* < 0.001, **** *p* < 0.0001).

All statistical analyses were performed with GraphPad Prism 8.0 (GraphPad Software, Boston, MA, USA). Differences among the three independent groups were evaluated using an ordinary one-way ANOVA, as shown in Figure 2, Figure 3 and Figure 4C,D. When significant effects were found, Tukey’s multiple comparisons test was used for post hoc pairwise comparisons. Two-way ANOVA was conducted to analyze the data in Figure 4B and Figure 5D. When significant effects were identified, Sidak’s multiple comparisons test was utilized for post hoc pairwise comparisons. For the ELISA results shown in Figure 5B,C, differences between the two groups were evaluated using an unpaired two-tailed Student’s *t*-test. The results are reported as the standard error of the mean (SEM) or standard deviation (SD), and a *p*-value below 0.05 was regarded as statistically significant.

## 3. Results

### 3.1. Ag-Specific Humoral and Cellular Immune Responses Were Induced by Intranasal Immunization with the Recombinant RBD Conjugate

We conjugated recombinant RBD19 of the SARS-CoV-2 spike protein with HBD2 and Co4B at the N and C-termini (329–521 a.a.), respectively (Figure 1). To evaluate the immunogenicity of the recombinant Ag conjugate, PBS, RBD19, and HBD2-RBD19-Co4B were administered intranasally to C57BL/6 mice, and the levels of Ag-specific IgG and IgA in serum and BALF were measured. The levels of Ag-specific IgG were significantly higher in the sera (*p* < 0.05) and BALF (*p* < 0.01) of the group immunized with the conjugate than in the group immunized with RBD19 alone (Figure 2A,B). Although the level of Ag-specific IgA, a major component of mucosal immunity, in BALF showed no significant difference among the groups, it was higher in the group immunized with the conjugate than in those immunized with PBS or RBD19 (1.36 ± 0.22 vs. 0.73 ± 0.09 or 1.14 ± 0.16 ng/mL) (Figure 2B). Additionally, the neutralizing activity against SARS-CoV-2 induced by the conjugate was significantly higher than that induced by PBS (*p* < 0.05) (Figure 2A). The focus-forming assay showed stronger inhibition of foci caused by SARS-CoV-2 infection in the BALF of mice immunized with the conjugate than in that from mice immunized with PBS or RBD19. However, the difference was not significant (Figure S1). These results indicate that a neutralizing Ab response to the virus can be effectively induced by a Ag construct containing the HBD2 and Co4B peptides.

Additionally, flow cytometric evaluation of Ag-specific cytotoxic T lymphocyte responses exhibited that the proportion of IFN-γ-producing CD8^+^ T-cells was significantly higher in the spleens of mice immunized with the conjugate compared to those in the other groups (Figure 2C). While the difference did not reach statistical significance, the proportion of IFN-γ-producing CD8^+^ T-cells in the lungs of mice immunized with the conjugate was generally higher than in mice immunized with PBS or RBD19 (0.59% ± 0.17% vs. 0.16% ± 0.03% or 0.33% ± 0.06%) (Figure 2C). Importantly, the persistence of the memory T-cell response in the lungs was confirmed 2 months after the final immunization. The proportion of CD44-expressing memory CD4^+^ T-cells was significantly higher in the conjugate-immunized group than in the PBS group (*p* < 0.01). It was also higher, although not significantly, than in the group immunized with RBD19 alone (Figure 2D). These results indicate that conjugating the recombinant RBD19 protein with HBD2 and Co4B can enhance systemic neutralizing Ab responses, Ag-specific cytotoxic T-cell responses, as well as Ag-specific IgG production and memory T-cell responses in the mucosa.

### 3.2. Intranasal Administration of Recombinant RBD Conjugate Elicited Protective Immune Responses Against SARS-CoV-2 in hACE2 KI Mice

ACE2 expressed in normal mice exhibits low binding affinity for SARS-CoV-2, resulting in inefficient viral infection [39]. Therefore, we utilized hACE2 KI mice, which are susceptible to SARS-CoV-2 infection, to assess whether the conjugate confers protection against viral challenge. First, we determined Ag-specific Ab and T-cell responses in hACE2 KI mice that received intranasal administration of each Ag protein, using the same schedule as in normal mice. Levels of Ag-specific IgG were significantly increased in both the sera (*p* < 0.01) and BALF (*p* < 0.01) of mice immunized with the conjugate compared to those immunized with RBD19 alone (Figure 3A). Although Ag-specific IgA levels did not differ significantly among groups, they showed an increasing trend in BALF from conjugate-immunized mice (Figure 3A). Furthermore, the proportion of Ag-specific GrB-producing CD8^+^ T-cells was significantly higher in the splenocytes and lung lymphocytes of mice immunized with the conjugate (*p* < 0.05) (Figure 3B). Additionally, the proportion of CD44-expressing memory CD4^+^ T-cells in the lung lymphocytes was significantly higher in mice immunized with the conjugate compared to those immunized with PBS or RBD19 (36.77% ± 1.30% vs. 17.47% ± 0.51%, *p* < 0.001, or 24.80% ± 0.77%, *p* < 0.01) (Figure 3C). These results suggest that the recombinant RBD19 protein conjugated with HBD2 and Co4B can enhance systemic and mucosal Ag-specific IgG production and memory T-cell responses in the lungs, which are necessary for inducing protective immune responses in hACE2 KI mice, an animal model of SARS-CoV-2 infection.

Next, we investigated whether the elicited Ag-specific immune responses could confer protection against SARS-CoV-2 challenge in mice (Figure 4). hACE2 KI mice were immunized with Ags according to the previously established schedule and then intranasally challenged with 2 × 10^5^ PFUs of SARS-CoV-2 (Figure 4A). For 8 days after SARS-CoV-2 infection, all mice survived and exhibited no signs of illness, such as body weight loss (Figure 4B). However, the abundance of the SARS-CoV-2 N gene transcripts in the lungs was significantly reduced in mice immunized with the conjugate compared to those immunized with PBS (*p* < 0.05). It was also lower than in mice immunized with RBD19 alone (Figure 4C). In addition, consistent with the results of the qRT-PCR expression assay for the viral gene identified in vivo, sera from the conjugate-immunized group displayed higher neutralizing Ab activity against SARS-CoV-2 than sera obtained from mice immunized with either PBS (*p* <0.001) or RBD19 (*p* < 0.05) (Figure 4D). These results indicate that the Ag protein conjugated with HBD2 and Co4B can provoke Ag-specific immune responses not only in the respiratory tract, the major route of infection, but also systemically, resisting infection with the virus challenge.

### 3.3. Siglec F Is a Functional Receptor That Interacts with the M Cell–Targeting Ligand Co4B

HBD2 is an AMP that binds to CCR2 and induces innate and adaptive immune responses through Nod2 signaling [30]. Previous studies have confirmed that Co4B can enhance adaptive immune responses [18,19], possibly due to interactions with unknown cellular components, such as M cell surface receptors. To identify the functional receptor that interacts with Co4B, we first searched the NCBI Protein BLAST database to find proteins homologous to Co4B. That search concluded that Co4B has 83% homology with amine oxidase copper containing 3 (AOC3), also known as vascular adhesion protein-1 (VAP-1) (Figure 5A) [40]. Interactions between VAP-1 and sialic acid–binding immunoglobulin-like lectins 9 and 10 (Siglec 9 and 10) are known in humans, but the same interactions have not been observed in mice [41,42]. However, it has been reported that Siglec F of the mouse Siglec (mSiglec) family is expressed on the surfaces of mouse M cells. There are also reports that human Siglec 5, which is functionally similar to mSiglec F, is expressed in human M cells [43]. We confirmed the interaction between Co4B and Siglec F using ELISA. EGFP-Co4B was produced and used to identify the functional receptors and mechanisms of interaction with Co4B. It showed higher binding affinity for plate-coated Siglec F than EGFP alone (*p* < 0.05) (Figure 5B). In addition, cross-validation confirmed that Siglec F binds more abundantly to plate-coated EGFP-Co4B than to plate-coated EGFP alone (*p* < 0.001) (Figure 5C). We further confirmed that the Co4B peptide alone can bind to Siglec F (Figure 5D) and that EGFP-Co4B administered intranasally binds to Siglec F, which is expressed in M cells present in NALT. The expression of Siglec F in M cells in the follicle-associated epithelium of NALT (Figure S2) and its co-localization with EGFP-Co4B were verified by CLSM (Figure 5E). Moreover, the interaction between EGFP-Co4B and Siglec F was observed in M cells in the PP (Figure 5F). These results suggest that Siglec F, which is expressed on M cells, is a functional receptor that specifically interacts with Co4B and effectively delivers Ags to the mucosal immune system. Further research is needed to elucidate the mechanism by which Siglec F interacts with Co4B.

## 4. Discussion

The protective lining of the respiratory tract, the primary site of infection, could serve as an effective site for preventing SARS-CoV-2 infection. Several vaccines targeting the respiratory tract, including mRNA-based vaccines, are being developed. However, further studies of key cells in the respiratory tract, which are also the route of vaccine administration, are needed to enhance vaccine efficacy [44,45,46]. In this study, we evaluated the effectiveness of a vaccine construct that combines two peptides, HBD2 and Co4B, with the RBD of the SARS-CoV-2 spike protein. Previous studies demonstrated that vaccine formulations using ligands targeting M cells in the mucosal lining can increase in vivo vaccine effectiveness [18,19]. Moreover, it has been shown that HBD2, an AMP from the mucous membrane, not only increases the innate immune response but also improves the adaptive immune response, thereby contributing to protective immunity against viruses [32]. External Ags that enter through the respiratory mucosa can be neutralized and eliminated by mucins secreted from the mucosal surface, which could reduce the effectiveness of vaccines administered via the nasal route. To address that challenge, we conjugated Co4B to the Ag to enhance mucosal immune response induction. Additionally, we used HBD2 to further boost mucosal immunity. However, it remains important to develop an appropriate formulation to improve the stability of Ag proteins and thereby enhance immune activation when they are used in the nasal route. Mucosal immunization with the conjugate (HBD2-RBD19-Co4B) effectively induced Ag-specific IgG and IgA responses in mice. Immunized mice showed significantly elevated Ag-specific IgG levels in both sera and BALF. Although the increase in the IgA response observed in the BALF of the conjugate-immunized group was not significant, the overall enhancement of T-cell responses in the lungs and neutralizing Ab activity in sera suggests that Ag-specific immune responses contribute to comprehensive protective immune responses. However, further studies are needed to enhance mucosal immunity, including the development of optimized mucosal formulations and the evaluation of diverse booster vaccination strategies.

We further investigated whether the induced Ag-specific immune responses were capable of protecting mice against a SARS-CoV-2 challenge. Because wild-type mice are not susceptible to SARS-CoV-2 infection, genetically engineered K18-hACE2 KI and hACE2 KI mice—modified to express the human *ACE2* gene—have primarily been used to investigate the pathological mechanisms of SARS-CoV-2 infection and assess the effectiveness of vaccines [36]. K18-hACE2 KI mice, engineered to express the *hACE2* gene under the K18 promoter, display elevated hACE2 levels in diverse epithelial cells and show pronounced morbidity and mortality. After infection with 1 × 10^5^ PFUs of SARS-CoV-2, several studies have observed mortality rates of 100% between days 5 and 7 and 75% between days 7 and 10 in these mice [34,47,48]. In contrast, hACE2 KI mice express hACE2 in the lungs and nasal turbinates and exhibit infection symptoms resembling those seen in humans, with relatively low morbidity and mortality [36]. Although variations in genetic design exist among hACE2 KI mouse models, those driven by the endogenous Ace2 promoter exhibit broadly similar ACE2 expression patterns and SARS-CoV-2 infection phenotypes, characterized by predominant lung infection and mild disease severity [49]. While infection resulted in greater concentrations of SARS-CoV-2 in the lungs and intestines of hACE2 KI mice compared to wild-type mice, the hACE2 KI model might be unsuitable for evaluating SARS-CoV-2 mortality [34,36,50]. In this study, we used hACE2 KI mice in our challenge experiments, and no deaths were observed; only mild symptoms during the early stages of infection. As a result, no notable differences in survival rates were seen among the immunized groups. However, at 8 dpi, mice in the conjugate-immunized group exhibited reduced SARS-CoV-2 gene transcripts in their lungs. These results indicated that mucosal delivery of the recombinant Ag protein conjugated with HBD2 and Co4B may lessen infection by the target virus. This study was conducted using the early Wuhan strain of SARS-CoV-2. Since then, the continuous emergence of variants, including multiple Omicron sublineages, has been reported. These emerging variants frequently contain additional mutations in the RBD, which may limit the effectiveness of immune responses elicited by variant-specific vaccines. Therefore, the rapid design of antigens incorporating updated RBD sequences that reflect circulating SARS-CoV-2 variants, combined with the application of two immunostimulatory peptides—Co4B and HBD2—could be a useful strategy to enhance variant-specific immunity. Furthermore, to achieve more durable and broadly protective immunity, combining this approach with multivalent vaccines containing diverse RBD sequences or cross-reactive strategies targeting conserved epitopes will be effective in countering newly emerging variants.

We aimed to identify the functional receptor that interacts with Co4B. The Siglec family consists of lectin proteins that bind to sialic acid, and the V-set immunoglobulin domain of Siglecs recognizes sialic acid–containing glycans [51]. Siglec F, which appears to bind Co4B, is primarily expressed in eosinophils and macrophages, but no reports have confirmed that the C2 domain of Siglec F interacts with functional proteins [51,52,53]. However, an interaction between the C2 domain and VAP-1 has been established for Siglecs 9 and 10, which are homologous to Siglecs E and G, suggesting a potential for binding between the C2 domain and functional proteins [41,42]. Siglec F contains an immunoreceptor tyrosine-based inhibitory motif (ITIM) and additional ITIM-like motifs within its intracellular domain, and is recognized as an inhibitory and apoptotic receptor. Nevertheless, cross-linking Siglec F in bone marrow–derived eosinophils robustly triggered the expression of pro-inflammatory genes and the production of type 2 cytokines, such as IL-4 and IL-13, as well as chemokines like CCL3 and CCL4, while having minimal impact on proliferation and apoptosis [54]. This enhancement in cytokine and chemokine secretion was abolished by removing the cytoplasmic tail of Siglec F [54]. Additionally, recent studies have reported that Siglec F is expressed in the M cells of PPs [43] and can induce endocytosis upon ligand binding [55,56]. This study also suggests that Siglec F is expressed on M cells in NALT and acts as a receptor for Co4B, an M cell–targeting ligand. Furthermore, the interaction between the Ag construct containing Co4B and Siglec F was confirmed by demonstrating the co-localization of Siglec F and HBD2-RBD19-Co4B within M cells of the follicle-associated epithelium in NALT (Figure S3). Therefore, we confirmed that Siglec F could be involved in Ag delivery and intracellular trafficking within M cells. Future research is needed to investigate the immunoregulatory mechanisms associated with its interaction with Co4B in the mucosal immune system. Furthermore, in this study, Siglec F was identified as a functional receptor for Co4B in mice; however, since it is not expressed in humans, the corresponding human protein remains unknown. Although human Siglec-8 exhibits functional similarity and may represent a translational analog, further validation is required.

Nasal vaccines have attracted attention as a potential strategy for preventing respiratory infections and preparing for future pandemics. However, olfactory epithelial cells in the nasal cavity—neurons that express olfactory receptors [57] and extend to the cerebrum via the olfactory nerve—pose the potential risk that intranasally administered vaccine Ags could affect the central nervous system [58]. Importantly, previous studies have evaluated the safety of nasally administered vaccines. Real-time tracking using ^18^F-labeled positron emission tomography–computed tomography and magnetic resonance imaging showed no accumulation of the intranasally delivered Ag in the cerebrum or olfactory bulb in either rodents or primates. Additionally, a real-time quantitative elimination profile of a nasally administered Ag was established, further supporting the efficacy and safety of nasal vaccines [59,60].

This study aimed to assess the effectiveness of a vaccine construct specifically designed to target M cells in the nasal mucosa. We showed that intranasal administration of a vaccine construct incorporating HBD2 and Co4B elicited adjuvant activity and induced enhanced mucosal and systemic immune responses against the virus. However, this study primarily evaluated short- to mid-term immune responses, and the long-term durability of immunity, including sustained mucosal protective and memory responses, remains to be determined. Moreover, using a formulation that enhances the stability of protein Ags is anticipated to increase both safety and efficacy when they are administered as a nasal vaccine in future studies. However, targeting M cells via Co4B is thought to enhance antigen uptake efficiency, thereby enabling the achievement of protective immune responses with lower doses or fewer vaccinations. Additionally, intranasal administration is expected to facilitate relatively easy multiple vaccinations. The advantages of mucosal vaccines-ease of administration, non-invasiveness, high patient compliance, and suitability for mass vaccination-are anticipated to promote the development of intranasal vaccines against respiratory viruses. However, intranasal administration of vaccines in humans presents practical challenges, including variability in dosing efficiency, inter-individual differences, and concerns regarding mucosal stability and safety, which should be addressed in future translational studies. Also, we showed that Co4B binds to Siglec F, which is expressed in M cells, indicating that the interaction between Co4B and Siglec F might enhance mucosal immune responses by facilitating the delivery and uptake of Ags conjugated with Co4B into mucosal immune tissues, such as NALT. In conclusion, these findings suggest that recombinant Ag constructs containing HBD2 and Co4B have potential as a vaccine platform for respiratory viruses.

## 5. Conclusions

This study demonstrated that Co4B facilitates the delivery and uptake of Co4B-conjugated antigens into the mucosal immune system by binding to Siglec F on M cells present in the mucosal immune tissues. To conclude, recombinant antigens conjugated with HBD2 and Co4B induced effective, though partial, antigen-specific humoral and cellular immune responses across both systemic and mucosal immune compartments. Thus, this recombinant antigen construct represents a mucosal vaccine platform with the potential to enhance both antibody and T cell immune responses against SARS-CoV-2.

## Data Availability

Data will be made available upon request.

## Supplementary Materials

The following supporting information can be downloaded at: https://www.mdpi.com/article/10.3390/vaccines14050421/s1. Figure S1: The levels of neutralizing Abs from C57BL/6 mice 2 months after they were immunized with recombinant RBD19 proteins, Figure S2: Expression of Siglec F on M cells in NALT, Figure S3: Interaction between Siglec F and HBD2-RBD19-Co4B on M cells in NALT, Figure S4: Gate setting strategy used for flow cytometry data analysis of lymphocytes obtained from C57BL/6 mice in Figure 2A and hACE2 KI mice in Figure 3B.
