# Supplementary material for: Mucosal Delivery of Recombinant SARS-CoV-2 Spike Receptor-Binding Domain Antigen Containing Immune-Stimulating Peptides Induces Protective Immune Responses Against Viral Infection in huACE2 Mice
Source: Vaccines (Basel). 2026 May 7;14(5):421. doi: 10.3390/vaccines14050421 (PMC13211405; doi:10.3390/vaccines14050421)
Supplement: Supplementary file 1 [file vaccines-14-00421-s001.zip › vaccines-4246708-supplementary.pdf]

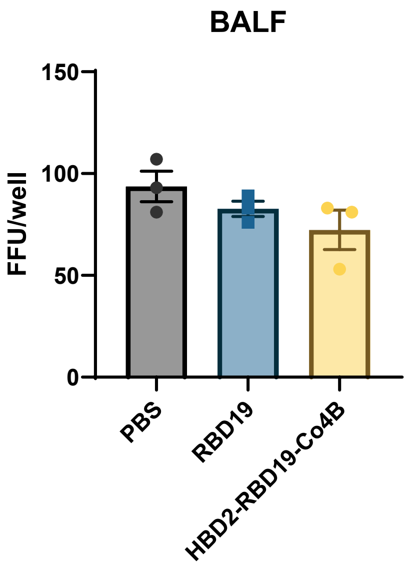

Figure S1. The levels of neutralizing Abs from C57BL/6 mice 2 months after they were immunized with recombinant RBD19 proteins. Initially, the virus was incubated with BALF obtained 7 days after last boost immunization, and was incubated with Vero E6. Foci were counted using a focus-forming assay, as described in the Materials and methods.

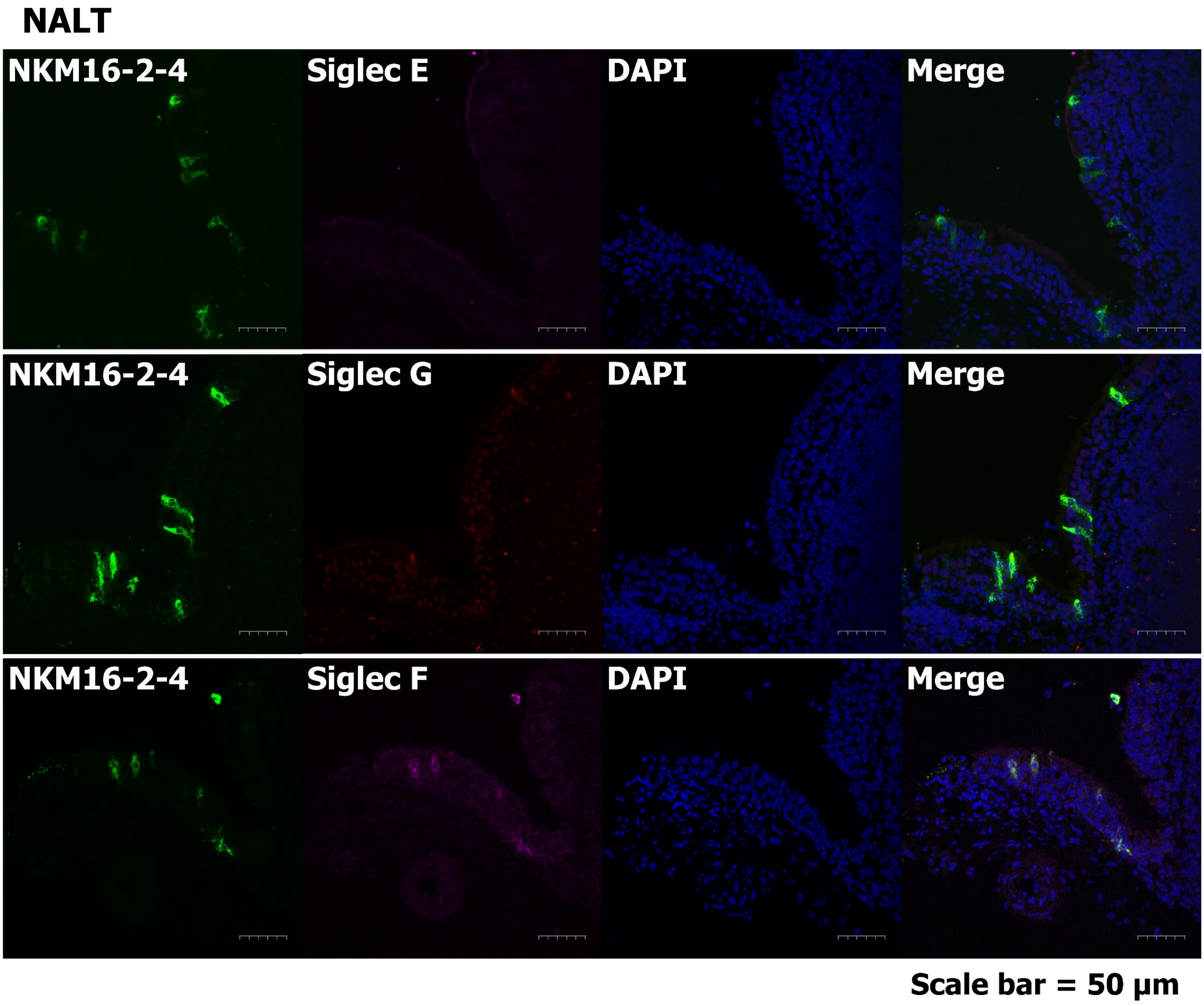

Figure S2. Expression of Siglec F on M cells in NALT. The expression of Siglec F (purple) on M cells (NKM16-2-4, green) was detected using an immunofluorescence stain.

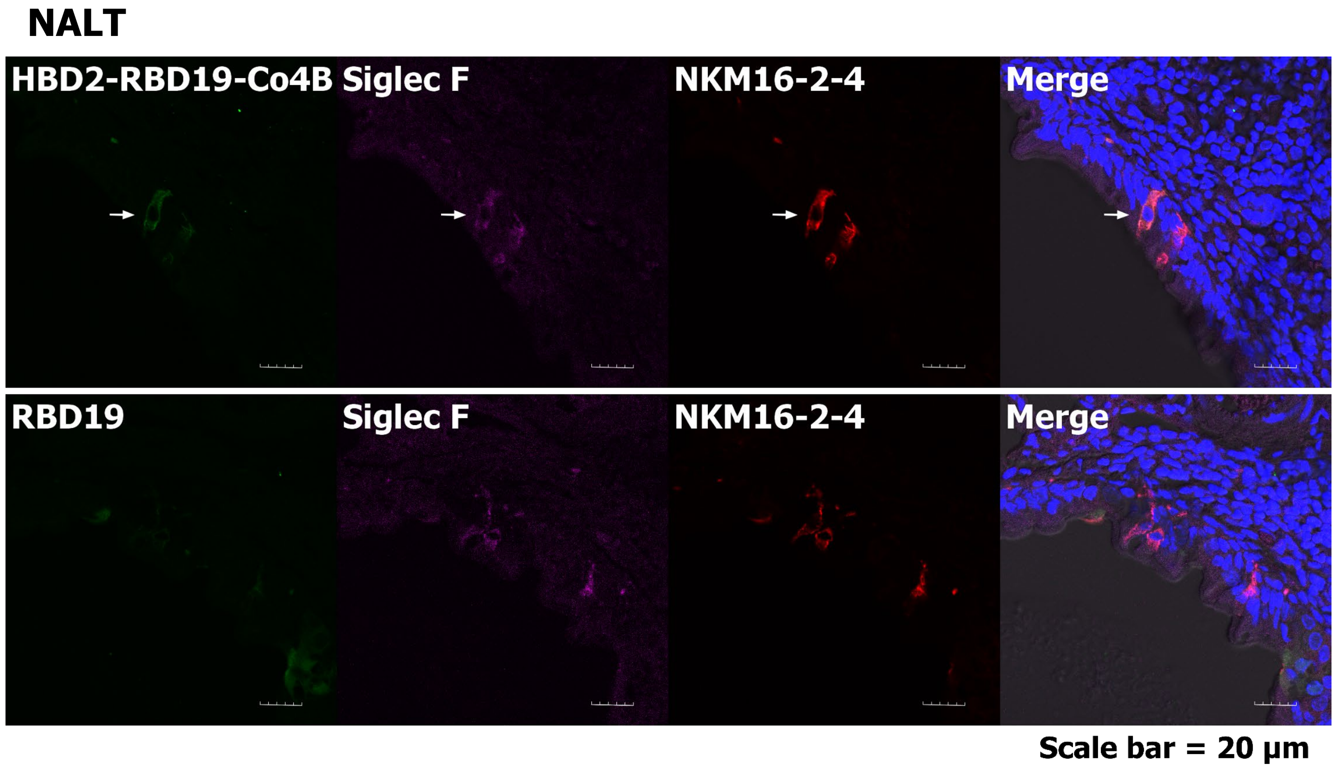

Figure S3. Interaction between Siglec F and HBD2-RBD19-Co4B on M cells in NALT. HBD2-RBD19-Co4B proteins (green) interacting with Siglec F (purple) on M cells (NKM16-2-4, red) was detected using an immunofluorescence stain.

(A)

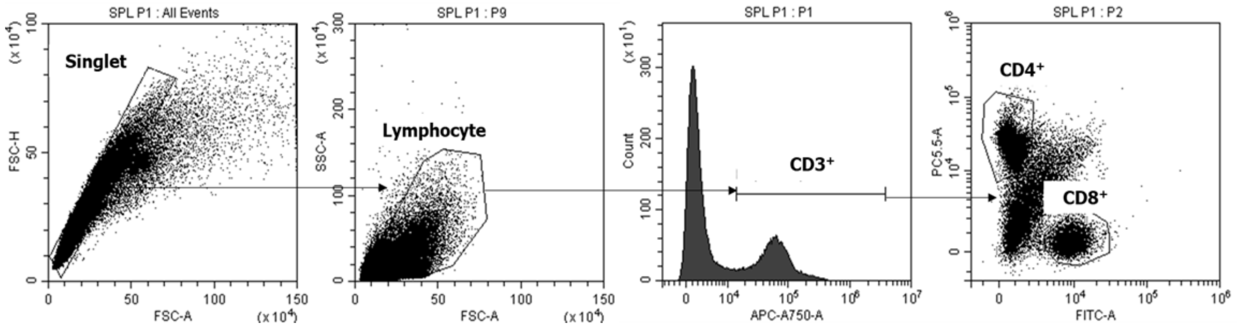

(B)

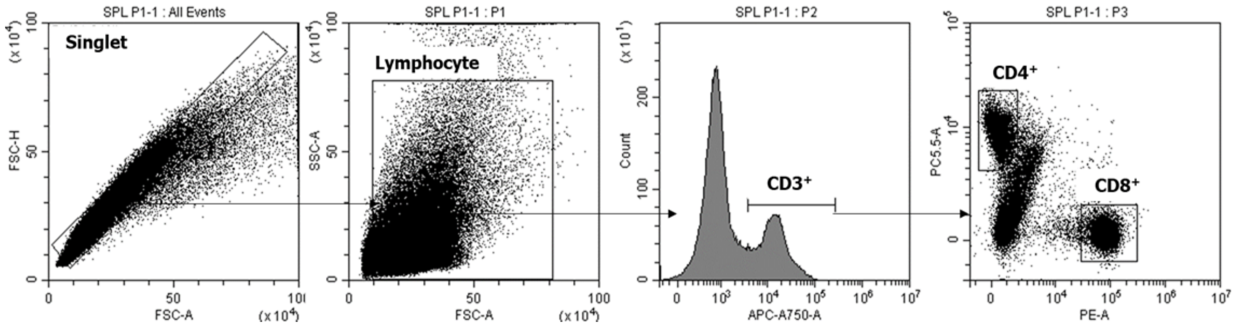

Figure S4. Gate setting strategy used for flow cytometry data analysis of lymphocytes obtained from C57BL/6 mice in Fig. 2 (A) and hACE2 KI mice in Fig. 3 (B).
